# Gendered dimensions of obesity in childhood and adolescence

**DOI:** 10.1186/1475-2891-7-1

**Published:** 2008-01-14

**Authors:** Helen N Sweeting

**Affiliations:** 1 MRC Social and Public Health Sciences Unit, 4, Lilybank Gardens, Glasgow, G12 8RZ, UK

## Abstract

**Background:**

The literature on childhood and adolescent obesity is vast. In addition to producing a general overview, this paper aims to highlight gender differences or similarities, an area which has tended not to be the principal focus of this literature.

**Methods:**

Databases were searched using the terms 'obesity' and 'child', 'adolescent', 'teenager', 'youth', 'young people', 'sex', 'gender', 'masculine', 'feminine', 'male', 'female', 'boy' and 'girl' (or variations on these terms). In order to limit the potential literature, the main focus is on other reviews, both general and relating to specific aspects of obesity.

**Results:**

The findings of genetic studies are similar for males and females, and differences in obesity rates as defined by body mass index are generally small and inconsistent. However, differences between males and females due to biology are evident in the patterning of body fat, the fat levels at which health risks become apparent, levels of resting energy expenditure and energy requirements, ability to engage in certain physical activities and the consequences of obesity for the female reproductive system. Differences due to society or culture include food choices and dietary concerns, overall physical activity levels, body satisfaction and the long-term psychosocial consequences of childhood and adolescent obesity.

**Conclusion:**

This review suggests differences between males and females in exposure and vulnerability to obesogenic environments, the consequences of child and adolescent obesity, and responses to interventions for the condition. A clearer focus on gender differences is required among both researchers and policy makers within this field.

## Introduction

The 'epidemic' of childhood and adolescent obesity has spawned a similar epidemic of research publications. These include many excellent reviews, produced for both clinical and epidemiological audiences (e.g. [1-3]). As an indication of the scale of the literature, Medline and Web of Science searches (September 2007) using the terms (or variations on terms) 'obesity' and ('child' or 'adolescent' or 'teenager' or 'youth' or 'young people') and limited to 1996–2007 English language publications, produced 11,280 and 9,067 hits respectively; addition of the term 'review' reduced the numbers to 1,991 and 552.

This paper reviews obesity in childhood and adolescence, but with a special focus on differences between males and females. These may result from differences in biology (sex differences) or those assumed to be due to society or culture (gender differences), or a combination of the two [4,5]. Both sex and gender are relevant to obesity. It is a product of, and impacts upon, both biology and behavior, and is associated with socially constructed attitudes and beliefs. Given the relevance of both sex and gender, a problem arises as to how to describe differences between males and females. To use the term 'gender difference' to describe purely biological differences is to misapply it [4], however to use 'sex' and 'gender' differences interchangeably because they cannot be entirely separated [6] is confusing and fails to address the issue. Here, the term 'gender' is used, since the majority of differences are unlikely to have solely biological origins.

Given this focus, databases including Medline, Embase, Web of Knowledge, Psychinfo and Cinahl were searched using the terms described above together with 'sex', 'gender', 'masculine', 'feminine', 'male', 'female', 'boy' and 'girl' (or variations on these terms). The aim was not simply to identify literature on differences between males and females, but also to investigate whether there was evidence of differences within what has been described as the 'gender/sex dichotomy' [5]. After reviewing the resulting titles and/or abstracts, it was decided to focus in the main on other reviews, both general and relating to specific aspects of obesity, in order to limit the vast potential literature. However, it should be recognized that reviews, particularly conventional narrative reviews, may be subject to errors in both data handling and reporting [7]. An exception to the use of review papers was made in respect of the sub-section on 'Distribution of obesity as defined by BMI', because to do so would have meant that results from individual studies of rates among males and females could not be presented.

The sections which follow this introduction describe definitions and distribution, causes, consequences and interventions for child and adolescent obesity. Gender differences, when identified, are highlighted.

## Definitions and distribution

### Definitions and measurement

Obesity can be defined as a pathological excess of body fat [1,2,8,9]. However, fat is not homogeneous, and abdominal fat has emerged as most clinically relevant [10]. Differences between males and females in adult body fat have long been recognized, the android, male, fat pattern represented by relatively greater fat in the upper body, the gynoid, female, pattern by relatively greater amounts in the hip and thigh areas [11]. Body fat distribution is partially hormonally controlled, and differences between males and females in fat mass have generally been considered to become manifest during puberty [1,12]. However, recent studies of pre-pubertal children have also found higher levels of fat and evidence of the gynoid pattern among females at much younger ages [13]. Studies which have assessed the degree of body fat associated with significantly elevated cardiovascular risk factors in children and adolescents, suggest that health risks become evident at lower levels for males than females [13].

Given its association with health risks, it has been suggested that there should be a greater emphasis than is current on the assessment of fat, particularly abdominal fat, for both clinical and epidemiological purposes [14]. However, the most accurate measures of body fatness are generally expensive, require laboratory conditions and/or are associated with reduced participant acceptability. Most epidemiological studies therefore rely on anthropometry, which can include measurements of skinfolds, or of waist and hip circumferences representing abdominal fat. The most frequently used measure is weight in relation to height, usually represented by the body mass index (BMI), defined as weight (kg)/height squared (m^2^) [13].

BMI has, despite many recognized limitations [15], been described as crucial to obesity classification and monitoring [14]. Given appropriate cut-offs, it can be used to classify obese children and adolescents with high specificity, thus identifying few false positives [16]. However, BMI varies according to both gender and age, and much less is known about specific BMI levels associated with health risks among children and adolescents than among adults [8]. BMI-for-age gender-specific reference charts with recommended cut-offs defining obesity have been developed in a number of countries. In addition, the International Obesity task Force (IOTF) has produced charts based on pooled international data using centile curves based on the recommended definitions of adult overweight and obese, aiming to compare across populations and employ a consistent definition throughout the lifespan [17-19]. Although the majority of studies adopt one or other of these definitions, alternative cut-offs continue to be used [20], but with decreasing frequency. The range of definitions means that it is often difficult to compare obesity rates between studies, because the methods used impact on the proportion of children defined as obese [13,21].

### Distribution of obesity as defined by BMI

Table 1, which shows obesity rates at several time points and in a large number of different countries, demonstrates the impact of different definitions.

**Table 1:** Examples of international obesity rates; different definitions, ages and dates – males and females

**Country**	**Survey/data**	**Definition**	**Age(s)**	**Date(s)**	**% males**	**% females**	**Reference**
North America							
United States	National Health and Nutrition Examination Surveys	US National Center for Health Statistics (1979) weight for length (children younger than 3) and weight for stature curves > 95^th ^percentile	< 1	1976–80	4.0	6.2	Ogden, Troiano & Briefel et al, 1997 [134]
				1988–94	7.5	10.8	
			4–5	1976–80	4.4	7.6	
				1988–94	5.0	10.8	
United States	National Health and Nutrition Examination Surveys	US Centers for Disease Control 2000 charts (CDC 2000) [135], >= 95^th ^percentile	6–11	1976–80	6.6	6.4	Baskin, Ard & Franklin et al, 2005 [136]
				1999–2002	16.9	14.7	
			12–19	1976–80	4.8	5.3	
				1999–2002	16.7	15.4	
Canada	Nationally representative surveys (1981 Canada Fitness Survey; 1996 National Longitudinal Survey of Children and Youth)	International Obesity Task Force (IOTF) [137] 'obesity'	7–13	1981	2.0	2.0	Tremblay, Katzmarzyk & Willms, 2002 [138]
				1996	10.0	9.0	
Trinidad and Tobago	Nationally representative cross-sectional schools-based survey	IOTF 'obesity'	5–6	1999	1.7	2.4	Gulliford, Mahabir Rocke et al (2001) [139]
			8–9		3.0	2.5	
Martinique	Schools-based surveys	IOTF 'obesity'	14	1999	6.3	4.8	Benefice, Caiius & Garnier (2003) [140]
South America							
Chile	School census data	IOTF 'obesity'	6	1987	1.8	2.1	Kain, Uauy & Vio et al, 2002 [141]
				2000	7.2	7.5	
		CDC 2000 >= 95^th ^percentile	6	1987	5.1	4.0	
				2000	14.7	15.8	
Mexico	Schools-based surveys	CDC 2000 >= 95^th ^percentile	11–14	1998–99	12.9	10.8	Salazar-Martinez, Allen & Fernandez-Ortega et al (2006) [142]
			15–19		8.0	4.9	
Australia							
Australia	National surveys (1985 Australian Health & Fitness Survey; 1995 National Nutrition Survey)	IOTF 'obesity'	7–11	1985	1.5	1.9	Magarey, Daniels & Boulton, 2001 [143]
				1995	3.7	6.3	
			12–15	1985	1.9	1.3	
				1995	6.1	4.4	
Europe							
England	National Study of Health and Growth (independent cross-sectional surveys)	IOTF 'obesity'	4–11	1974	1.4	1.5	Chinn & Rona, 2001 [144]
				1994	1.7	2.6	
Scotland				1974	1.7	1.9	
				1994	2.1	3.2	
England	Health Survey for England	United Kingdom (UK90) charts [145] >= 95^th ^percentile	2–10	1995	9.6	10.3	National Health Service Information Centre, 2006 [146]
				2004	16.2	11.9	
			11–15	1995	13.5	15.4	
				2004	23.7	26.2	
Scotland	Scottish Health Survey	UK90 >= 95^th ^percentile	12–15	1998	15.6	15.2	Scottish Public Health Observatory, 2007 [147]
				2003	20.9	14.7	
Northern Ireland	Schools-based surveys	IOTF 'obesity'	12	1989–90	4.0	1.6	Watkins, Murray & McCarron et al, 2005 [148]
				2000	4.7	4.7	
			15	1989–90	0.4	3.9	
				2000	3.1	4.8	
France	Schools-based surveys	IOTF 'obesity'	7–9	2000	3.9	3.6	Rolland-Cachera, Casetbon & Arnault et al, 2002 [149]
		CDC 2000 >= 95^th ^percentile			7.5	5.2	
Spain	Cross-sectional surveys in randomly selected schools	IOTF 'obesity'	6–14	1980	1.5	1.4	Moreno, 2001 [150]
				1995	2.1	3.3	
Portugal	Schools-based surveys	IOTF' obesity'	7–9	2002–03	10.3	12.3	Padez, Fernandes & Mourao, 2004 [151]
Italy	Schools-based surveys in North (N) and South (S) Italy	IOTF 'obesity'	2–6	2002	5.7 (N)	5.8 (N)	Maffeis, Consolaro & Cavarzere et al, 2006 [152]
					12.3 (S)	10.7 (S)	
Sicily, Italy	Schools-based surveys	IOTF 'obesity'	11	1999–2001	13.1	10.7	Baratta, Degano & Leonardi et al, 2006 [153]
			15		7.2	5.0	
Greece	Schools-based surveys	IOTF 'obesity'	6–11	1994	9.4	8.3	Papadimitriou, Kounadi & Konstantinidou et al, 2006 [154]
				2005	12.3	9.9	
Greece	Schools-based surveys	IOTF 'obesity'	7–10	2002–03 *	16.0	13.4	Tokmakidis, Kasambalis & Christodoulos, 2006 [155]
Finland	Nationally representative Adolescent Health and Lifestyle Survey	IOTF 'obesity'	12	1977	2.0	0.9	Kautiainen, 2005 [156]
				2003	3.1	1.9	
			16	1977	0.8	0.3	
				2003	4.2	2.1	
Denmark	Nationally representative samples	IOTF 'obesity'	14–16	1971–72	0.4	0.9	Kautiainen, 2005 [156]
				1996–97	2.0	2.6	
East Germany	Schools-based surveys	IOTF 'obesity'	5–7	1992–93	4.7	3.5	Frye & Heinrich, 2003 [157]
				1998–99	2.7	4.8	
			11–14	1992–93	2.8	3.5	
				1998–99	7.1	7.9	
Poland	Schools-based surveys	IOTF 'obesity'	7–9	2001	3.6	3.7	Malecka-Tendera, Klimek & Matusik et al, 2005 [158]
Russia	Nationally representative household survey	IOTF 'obesity'	6–9	1992	9.5	11.2	Wang & Wang, 2002 [159]
			10–18		2.4	1.3	
		US Must, Dallal & Dietz 1991 (MDD) charts [160], >= 95^th ^percentile	6–9		13.2	15.9	
			10–18		3.1	1.6	
Russia	Nationally representative household surveys	CDC 2000 >= 95^th ^percentile	7–13	1995	6.2	3.0	Tudor-Locke, Ainsworth & Popkin, in press [161]
				2002	4.2	3.1	
Africa							
Egypt	Schools-based surveys	CDC 2000 >= 95th percentile	11–14	1997	6.6	7.6	Salazar-Martinez, Allen & Fernandez-Ortega et al, 2006 [142]
			15–19		5.9	8.6	
Senegal	Survey of members of birth cohort	IOTF 'obesity'	12–17	1998–2000	0.0	0.0	Benefice, Caiius & Garnier, 2003 [140]
South Africa	Primary School Surveys	IOTF 'obesity'	8	1994–95	0.4	0.6	Jinabhai, Taylor & Sullivan, 2003 [162]
Asia							
Saudi Arabia	Cross-sectional survey of children from randomly selected households	IOTF 'obesity'	1–18	1994–98	6.0	6.7	El-Hazmi & Warsy, 2002 [163]
Qatar	Schools-based surveys	MDD >= 95^th ^percentile	7	2002	1.6	5.4	Qotba & Al-Isa, 2007 [164]
Bahrain	Schools-based surveys	IOTF 'obesity'	12–17	2000	14.9	17.9	Al-Sendi, Shetty & Musaiger, 2003 [165]
		MDD >= 95^th ^percentile			16.5	20.2	
Japan	Annual cross-sectional household surveys	IOTF 'obesity'	6–8	1976–80	1.8	1.8	Matsushita, Yoshiike & Kaneda et al, 2004 [166]
				1996–2000	4.6	4.6	
			12–14	1976–80	1.0	0.5	
				1996–2000	2.7	1.0	
Korea	National survey	>120% ideal body weight	0–19	2001	15.1	10.2	Kim, Ahn & Nam, 2005 [167]
Taiwan	Schools-based surveys	>120% ideal body weight	12–15	1980–82	12.4	10.1	Chu, 2001 [168]
				1994–96	16.4	11.1	
China – Beijing	Schools-based surveys in Beijing (B) and Shanghai (S)	BMI classification reference provided by Working Group on Obesity in China	7–18	1985	1.2 (B)	1.1 (B)	Ji, 2007 [169]
					1.1 (S)	0.4 (S)	
				2000	10.0 (B)	5.2 (B)	
					7.2 (S)	4.1 (S)	
China	Schools-based surveys	IOTF 'obesity'	13–14	2002	2.6	1.5	Xie, Chou & Spruijt-Metz et al, 2007 [170]
			16–17		4.3	1.0	
China	Schools-based surveys	IOTF 'obesity'	11–17	2004	5.0	2.1	Li, Dibley & Sibbritt et al, 2006 [32]
Punjab, India	Schools-based surveys of children from affluent families	IOTF 'obesity'	6–11	2002 *	5.9	6.3	Sidhu, Kaur & Kaur, 2006 [171]
India	Schools-based surveys	Triceps skinfold thickness >= 90^th ^percentile	9–15	1998 *	12.4	9.9	Chhatwal, Verma & Kaur, 2004 [172]

* survey dates provided by authors (personal communication).

Despite different definitions, the wide range in prevalences between countries is clear. This was also seen in a study of European, Israeli and US adolescents, who self-reported height and weight in 1997–98. These data are not shown in Table 1 since the definition was not 'standard', being the 95^th ^BMI centile for age and gender derived for the whole sample. Overall rates were therefore around 5%, with the lowest rates in Lithuania, and the highest in the United States [22]. Very similarly, a comparison of obesity prevalence, based on the IOTF definition among adolescents from 34 countries who self-reported height and weight in 2001–02 found the lowest rates in Lithuania and Latvia, and the highest in Malta and the United States. This study noted particularly high prevalences in countries in North America, Great Britain and south-western Europe [23]. A review of surveys conducted within Europe during the 1990s, suggested higher levels of childhood overweight and obesity in southern countries and lower levels in the central and eastern countries which experienced political and economic transition over that period [24]. Within developing countries, high prevalences are found in the Middle East [25].

Returning to Table 1, a further issue demonstrated is that of globally increasing rates, regardless of definition. Obesity has been described as the most common pediatric disease in most of the world, with the exceptions of sub-Saharan Africa and the former Soviet Union [26]. Within Europe, the prevalence rates of overweight and obesity are not rising at a constant rate, but are accelerating [27]. In respect of secular changes, what Table 1 cannot show is that in addition to increases in obesity rates, the distribution of BMI has become more skewed, with the greatest increases at the highest levels [28,29]. There is also evidence of changing body composition, with increasing fat and reductions in muscle [30,31].

In respect of gender, Table 1 suggests no consistent male or female excess. Differences are generally small, and there is no suggestion that either males or females consistently predominate within particular age groups, or according to particular definitions. Although international patterns are, in general, also difficult to discern, Table 1 suggests that within Asia, a male excess may exist in Korea, Taiwan and China, in contrast to a female excess in Middle Eastern countries. A proposal that the male excess in China may result from gender differences in child care associated with the one-child policy [32], has been challenged [33]. An apparent female excess in some UK studies using the IOTF definition is artefactual, since this definition has been shown to have lower sensitivity in UK males than females [34]. The study which derived 95^th ^BMI centiles for age and gender within its international adolescent sample found higher rates among males in some countries, while in others there was a female excess. However, in almost all cases the gender differences were not statistically significant [22]. Similarly, the review of surveys conducted within Europe in the 1990s found the number of countries with higher prevalences for overweight and obesity among females was almost the same as that with higher prevalences for males [24]. It is possible that gender differences may emerge in future; a study conducted in the US found significantly greater increases in rates among males between 1986–98 [35]. It has therefore been suggested that prevalence estimates for both males and females should always be presented [2].

While gender differences may be small overall, patterns may differ according to ethnic group. Among females, British studies almost always find the highest rates of obesity among black females, with the lowest levels generally found among South Asians [36,37]. Among a sample large enough to sub-divide into black African and black Caribbean groups, one UK study of 11–13 year olds found higher BMIs among both groups than in their white, Indian, Pakistani, Bangladeshi or mixed ethnicity peers [38]. These studies have generally found smaller, and less consistent ethnic differences in obesity rates among males. However, a US study found the same pattern among both males and females, the highest rates occurring in Mexican Americans at ages 6–11, and Non-Hispanic blacks at ages 12 to 17 [39]. Different results in respect of rates according to ethnicity by gender in studies conducted in the UK and US may result in part from the different groups included; there are few US data on the health and weight status of Asian Americans [40].

In respect of socioeconomic status (SES), studies suggest higher rates of obesity among low income groups in richer countries, and high income groups in poorer ones [1,41]. It is suggested that this is because in developing nations, higher SES individuals have become globalized, with easy access to relatively cheap, calorie-dense foods, while those of lower SES remain localized and undernourished [42]. Within the US, increases have also been greatest among children and adolescents from the lowest income families, so increasing SES disparities [35]. However, not all studies in developed countries find SES differences [36,43,44], and among those that report separately, there is some evidence that SES differences may be clearer among females than males [37,45].

## Causes

### Genetic factors

Like height, weight is heritable. One recent review suggests that twin and adoption studies point to a genetic contribution for BMI of 40–70% [46], while a more extensive, but earlier, review of familial resemblance suggests that genetic factors explain 50–90% of BMI [47]. The results of genetic studies, where presented separately for males and females, appear broadly similar. Overall, the findings of such studies mean that genetic factors determine individual susceptibility to gain weight. Such 'thrifty' genes provide an evolutionary advantage in times of famine, when humans have to stockpile energy to survive, but a disadvantage when food is plentiful [48,49]. However, as several authors point out, while the propensity for obesity may have existed for a long time, the recent rapid rise in rates demonstrates the central role of environmental factors [46,47,50].

### Behavioral factors

Obesity is related to an imbalance between energy input and output, the size of which may be very small if over a long period [51]. One review suggests that in children, an imbalance of around 2%, which is the equivalent of around 30 calories or 15 minutes of tv instead of play a day, may lead to obesity [52]. Behavioral determinants therefore include excess energy intake and/or inadequate energy expenditure [53], although the emphasis given to these 'Big Two', and the neglect of other plausible contributors to the secular increase in obesity has recently been questioned [54].

In respect of energy intake, dietary surveys do not suggest a secular increase among children and adolescents. However, the results of such studies may be confounded by an increasing trend towards greater under-reporting, to the extent that reported intake may be below the estimated required physiological minimum, especially among older girls. Regular consumption of high energy-dense fast-foods and sugary drinks which are associated with less satiation and so insufficient compensation via subsequent reductions in intake, increased portion size, eating outside the home and snacking have been particularly implicated in promoting weight gain. This is especially the case among older children, who are less influenced by biological cues of satiety [50,55]. In respect of energy expenditure and physical activity, assessment is difficult [56], and evidence for secular trends is scarce because of the absence of suitable baseline data [57]. However, a decline in UK adolescent energy intake from the 1930s to the 1980s with no concurrent change in body mass, increasingly pervasive electronic and screen-based entertainment, greater car travel and reduced walking or cycling, together with evidence of reductions in fitness in developed countries all point to lower activity levels [57-60].

Gender differences in many areas of nutrition emerge in childhood and adolescence. The greater fat-free mass of males, particularly post puberty, requires a higher energy intake. Thus the US Government suggests approximate daily requirements for moderately active 4–8 year olds as being 1,500 kcal for both males and females, compared with 2,600 kcal for males and 2,000 kcal for females aged 14–18 [61]. Studies of adolescents have found that females are more likely to pay attention to foods as a way to influence health and to meet nutritional recommendations, while males eat more fast foods. It has been suggested that these differences arise not only because of differences in Western societies' perception of ideal body weights, but also because some foods are gendered; for example meat may represent strength and virility. In addition, the menstrual cycle has been associated with cravings for foods rich in fat and carbohydrate [12,62].

Gender differences in energy expenditure can also be attributed to both biological and social factors. Total energy expenditure (TEE) is strongly correlated with body weight, largely because of the relationship between resting energy expenditure and body weight [63]. Differences in body composition at puberty are associated with higher TEE among males than females, however there is limited and inconsistent data on male-female differences in TEE pre-pubertally [63,64]. Of all the components of TEE, physical activity is most amenable to modification [50], and a review of prospective observational studies has concluded that increased physical activity and decreased sedentary behavior protect against relative weight and fatness gains in childhood and adolescence [59].

It has been suggested that the clearest biological correlate of physical activity in children and adolescents is gender [65], and also that the effect of energy expenditure on the etiology of obesity may vary by gender, ethnicity or age [51,52,63]. Gender differences in physical activity begin in childhood. A longitudinal study found that while boys' daily TEE increased continuously between ages 5 and 10, that of girls increased from around 1,400 kcal at 5 to 1,800 kcal at 6, but by age 9 had reduced to 1,600 kcal. This reduction was explained by a 50% reduction in physical activity between ages 6 and 9 [52]. One review cites data suggesting that levels of moderate or vigorous physical activities in US 11–12 year old boys are nearly twice those of girls, while age-related decreases in activity levels in adolescence are higher in girls. Thus a 1990 UK study found decreases between ages 11 and 18 in mean self-report daily activity causing at least slight breathlessness from 83 to 57 minutes among males and from 49 to 26 among females. A review of the world literature suggested annual declines in physical activity of up to 2.7% among boys and 7.4% among girls between the ages of 10 and 17 [56,65,66]. Such findings have been related to differences in motor skills development, body composition, socialization towards sports and physical activity and freedom to engage in activities independently outside the home [56,65].

Sedentary behavior has also been associated with body composition and BMI. For example, children who watch more tv have been shown to have higher skinfold thicknesses, while one study has suggested that approximately 17% of early adult overweight may be attributable to watching tv for two or more hours daily in childhood (reported in [57]). Given this, it is interesting that a review of studies of screen-based media use in contemporary youth, most from Europe or the US, found that males are also higher users of tv and video games. For example, 30% of males and 25% of females watched tv for more than four hours a day, while 30% males and 7% females played video games for more than four hours a week. This suggests that females may spend time in sedentary activities not measured by most current instruments [67].

### Parental influences

Obesity clusters in families, and parental obesity is the most important risk factor for obesity in children [68]. While implicating genetic factors, the environments of children and, to a lesser extent, adolescents, are also generally provided by their parents [69,70]. There is evidence that familial patterns of adiposity may be partly attributable to similarities in diet. There is also evidence that self-regulation of energy intake by infants and children can be over-ridden by parental control, first among those who are formula fed and who may be encouraged to finish the bottle, then as well-meaning parents attempt to restrict children's eating. Gender differences have also been reported here: maternal restriction of snacks has been found to promote their over-consumption in an unrestricted setting by females, but not males; greater parental control has been linked to increased adiposity in females, but not males; mothers who use more control have been found to have daughters, but not sons, with poor energy regulation [69,71,72]. In addition, there is some evidence that parents are less likely to encourage sons to lose weight, perhaps because of the larger, more muscular ideal male body shape [73]. Parents also appear to be strong influences on physical activity in childhood, and again, there is evidence of gender differences; for example, stronger relationships with parental activity for girls [65,74].

## Consequences

Several reviews summarize the consequences, both short- and long-term, of childhood and adolescent obesity.

### Physical health consequences

Childhood physical health consequences were, until fairly recently, largely unrecognized. In fact, there are few organ systems that severe obesity does not affect [1]. Associated outcomes include: cardiovascular risk factors, identified in children as young as 5 years; type 2 diabetes; non-alcoholic fatty liver disease; asthma; sleep-disordered breathing; systemic inflammation; and orthopedic problems. There is a marked tendency for obesity, particularly adolescent obesity, to track into adulthood, and so be associated with adult obesity-related disorders. However, even after controlling for current risk factors including weight, childhood obesity is associated with increased risk of all-cause and coronary heart disease mortality [1,2,75-80].

Some of the physical consequences of obesity are gendered, with some evidence that adult mortality risks are higher in respect of adolescent obesity among males [75,76]. There have been suggestions of a stronger relationship between obesity and asthma in females, but results in this respect are inconsistent [81,82]. Among females, obesity is associated with early onset of puberty and menarche which have, in turn, been linked to breast cancer, although the relationships are complex [83]. Obesity has also been related to other cancers of the female reproductive system, increased risk of spontaneous abortion and menstrual problems, particularly polycystic ovary syndrome which can lead to infertility [1,11,12,49,68,84]. The relationship between obesity and female puberty led to the suggestion that a threshold level of fatness is required for the female growth spurt and menarche. This is not now thought to be the case [85,86]; indeed, there is a growing body of evidence suggesting the opposite, that puberty effects levels of fatness [15].

Links have also been suggested between increasing levels of obesity, its association with female puberty and claims of recent reductions in age of menarche. Reviews conflict, some suggesting 'clear evidence' that the age of menarche is falling [87] and others that while menarche may be stable, the first signs of puberty, such as breast budding may now be occurring earlier [85,86]. However, it may be the case that there has been no recent secular change in either, but rather that obesity is associated with premature development of pubic hair and apparent breast tissue separate from true puberty [88].

Since markers of puberty are more overt and recordable for females, there is less information about its timing for males [87]. However, some reviews of obesity and puberty appear to extrapolate the female findings to males or children/adolescents in general [68,85]. In fact, although the evidence is much sparser, there is some evidence that in males, obesity is associated with later sexual maturation [1,15,86].

### Psychosocial consequences

The immediate psychosocial consequences of childhood and adolescent obesity have been long-recognized [89]. Stigmatization and discrimination by peers is well-documented; from very young ages, obese children are characterized in negative ways, less preferred as friends and more likely to be the targets of teasing or bullying [1,2,75]. There is also evidence of bias and stereotyping by teachers and even some parents [90]. While it could be argued that such behaviors might diminish as obesity becomes more commonplace, this does not appear to have occurred [1].

Evidence in respect of the psychological consequences of child and adolescent obesity is mixed [1]. While some reviews conclude that it is associated with psychological or psychiatric problems, increasing with age and particularly among girls [78], others suggest that research has failed to find consistent differences in the global self-esteem [91], depression or anxiety [92] of obese or overweight children and adolescents compared with the rest of the population. Such conflicting results may have arisen in part because obese clinic samples tend to have poorer psychological well-being and quality of life than community samples. While obese community samples have lower body satisfaction and physical self-competence than non-obese, few are depressed or have low global self-esteem [93,94]. Small differences in their psychological well-being may also be explained, at least partly, by weight-related teasing or bullying [95]. There have been suggestions of associations between depression and obesity, and that major depression in adolescents, or young females, predicts increased adult BMI [95,96]. However, the relationship between obesity and depression has been described as unproved [94].

Reductions in body satisfaction and well-being amongst the obese are greater among adolescents than children, and among females than males. Reflecting this, 12 of 18 recent studies of body dissatisfaction and 7 of 28 of self-esteem reported in one review included female participants only [94]. The consensus is that gender differences in body satisfaction emerge around 8–10 years of age [73]. While both males and females with a high BMI wish to be thinner, the picture is more complex for males because of the larger muscular male ideal. In relation to stereotypes and attitudes, some studies have demonstrated equivalent stereotyping of obese peers, regardless of gender. However others have suggested that females express stronger dislike of obese peers than males, and that obese females are rated more negatively than obese males. There is also some evidence that females may be more vulnerable to obesity-related victimization, but again, this is not a consistent finding [90,97]. The picture in relation to body satisfaction and obesity is also complicated by ethnic differences. For example, black girls with high BMIs are less likely than those from other ethnic groups to consider themselves overweight or desire to be thin [90,98].

Gender differences in weight-control methods are also evident. Males are more likely to exercise, begin dieting at a higher BMI than females, and tend to focus on increasing upper body size while reducing fat [99]. In contrast to professionally administered, 'sensible' weight loss programs, [100], the use of unhealthy quick-fix dieting practices by some adolescents has been linked to a range of negative physiological and psychological outcomes [101,102]. Although such behaviors are significantly more likely among females [102,103], males are not immune to disordered eating behaviors [99,104]. The finding of one study that femininity as represented by self-ascribed expressive personality traits, was predictive of eating problems among both male and female adolescents [105], highlights the need to consider such characteristics within each gender, rather than just compare the behaviors of males and females.

Finally, to return to the longer-term consequences, longitudinal studies in both the US and UK have demonstrated that child and adolescent obesity is associated with adverse social and economic outcomes such as reduced years of education, income and marriage rates in young adulthood. Again, there are gender differences, these effects being stronger for females [1,2,75,76,78].

## Interventions

The goal of any weight loss intervention is to achieve an energy imbalance so that intake is less than expenditure [77]. Most therefore target food consumption and/or physical activity either directly or indirectly [106]. A large number of reviews are now available, all focusing on the same, surprisingly small, evidence base. The majority conclude that: (a) treatment or prevention efforts have some positive effects, although these are generally small or very small; (b) a global approach, including various methods and settings, is likely to be more effective; and (c) given proper implementation and monitoring, there is little evidence of negative effects, either physiological or psychological. [70,77,80,100,106-122]. While some reviews have been unable to identify any particular interventions that characterize the positive studies [118], others have suggested that effective interventions can be distinguished on the basis of such factors as 'compulsory' aerobic physical activity [123], parental involvement [124] or more motivated participants [121,125]. Some authors have been more optimistic about the treatment of pediatric, as opposed to adult obesity, because of factors such as family support, and the fact that weight stabilization, rather than reduction, may be sufficient. Despite this, most pediatric obesity interventions are marked by relapse, although there is some evidence for long-term efficacy [107].

In respect of approaches, the most common include diets such as the traffic light diet (foods categorized as 'go', 'caution' and 'stop'), exercise sessions and increased general lifestyle activity, combined with the use of behavior change methods. For example, since obese children have a more negative perception of physical activity than non-obese children, and also find activity less reinforcing than sedentary behaviors, rewarding reductions in sedentary behaviors in order to increase physical activity has been found to be effective [107].

In respect of setting, a recent American Dietetic Association (ADA) position paper, based on a systematic evidence-based approach to the literature [106], categorized pediatric obesity interventions into three types; tertiary, secondary and primary, based on their target populations (the obese, those at risk of obesity and those with lower BMIs). In respect of tertiary prevention, limited evidence was found for individual-based interventions, but there was evidence that family-based interventions should be routinely recommended for 5–12 year olds, although less evidence to support similar recommendations for adolescents. School-based programs were recommended for primary prevention, and although equally effective for secondary prevention, were not recommended due to the risk of stigma among those targeted and because continually increasing rates of obesity suggest the need for population-wide approaches. Despite insufficient evidence to evaluate community-based approaches (for an extensive list of such policies see [126]), such interventions were recommended due to their potential to impact on the greatest numbers [106].

The ADA paper identified no studies which evaluated the efficacy of popular weight loss approaches, such as programs via the internet, in self-help formats or non commercial settings [106]. The internet has been identified as a potentially powerful tool for obesity intervention for a number of reasons, including its anonymity and accessibility. It has been suggested that it may be particularly successful among adolescents, given their high rates of internet use [127].

The ADA paper did not consider gender differences, however another recent review of the literature on the prevention and treatment of childhood obesity did [80]. Importantly, from the perspective of the current review, one of the gaps identified was that, along with immigrant and minority ethnic groups, and those aged below six years, males represented a population subgroup where obesity prevention programs and evidence of effectiveness were limited. In addition, it was noted that few programs were gender-specific.

There are a number of reasons why obesity prevention programs are more likely to be developed for girls, particularly adolescent girls [80]. As noted earlier, concerns about body image and vulnerability to eating disorders are greater, and participation in physical activity lower, among girls. However, this may have blinded those working in this field to the fact that the growing prevalence of obesity affects males and females equally. Further, gender-specific programs may be more effective than blanket approaches; some trials have found different effects for males compared with females [77,115,128]. Such evidence as there is suggests that males respond better to physical activity interventions [125]. It has been suggested that gender differences in the effects of interventions may become more apparent in adolescence, as biological and environmental differences increase [129,130].

Despite such findings, a recent review of RCTs of exercise for treating obesity in children and adolescents found that not all trials described gender characteristics, and many which did reported combined results for males and females. Further, between-group differences in gender distribution and maturation stage tended not to be considered, despite their potential confounding effects on obesity outcomes [131]. Ignoring potential gender differences in effectiveness might result in failure to identify successful programs [122], while investigation of different effects for males and females might increase understanding of mechanisms of behavior change and optimize interventions [132].

## Conclusion

Although not generally the principal focus of the literature on childhood and adolescent obesity, gender is a thread running through much of it. This review has highlighted the ways in which differences between males and females, both biological and those due to society or culture are relevant to obesity in childhood and adolescence. While the findings of genetic studies are broadly similar for males and females, and differences in obesity rates as defined by BMI are small and inconsistent, biological differences between males and females are apparent in the development of fat patterning and the association between levels of fat and health risks. Biological differences are also evident in respect of the female-limited consequences of obesity.

Biological and social factors merge in the development and impact of obesogenic behaviors, the long-term physical health consequences of the condition and the success of certain interventions. The greater fat-free mass of males is associated with greater energy expenditure and requirements, and differences between the bodies of males and females may impact on abilities to engage in certain physical or sporting activities. However, gender differences in food choices and dietary concerns, as well as those in overall physical activity levels are the result of social factors. Culture-bound conventions and roles determine these behaviors, with societal expectations and stereotypes for males and females being transmitted via parental, peer and media influences. Finally, social, rather than biological factors are evident in respect of gender differences in body satisfaction and the long-term psychosocial consequences of childhood and adolescent obesity. Being thin is highly valued in our Western society, but more so for females for whom it is associated with beauty, and poorer social or economic outcomes for obese females suggest gendered stigmatization and prejudice.

This review has also suggested that the patterning of child and adolescent obesity according to ethnicity or SES may differ for males compared with females. This highlights the importance of reporting not only overall prevalences, but also the socio-demographic patterning of obesity separately for males and females or, alternatively, noting that gender differences are absent. Studies which fail to do this, or which extrapolate from one gender to the other, may obscure differences which might help explain the distribution of obesity. One aim of this review was to identify gender differences within, as well as between males and females, for example in respect of masculine and feminine traits associated with obesity or behaviors. With one or two exceptions [97,105], it appears that almost no attention has been paid to this area.

In sum, the evidence reviewed here suggests differences between males and females in both exposure and vulnerability to obesogenic environments, as well as in the consequences of obesity and responses to interventions. In doing so, it highlights the need for a clearer focus on gender differences within the field of childhood and adolescent obesity among both researchers and policy makers. To date, work in this area has tended to assume that the biological and social correspond [133], thus polarizing males and females. However, in relation to areas such as body image, self-esteem, dietary and exercise behaviors and interventions, it may be valuable to go further and to consider the impact of masculinity and femininity among both males and females.
